# The superior photocatalytic performance and DFT insights of S-scheme CuO@TiO_2_ heterojunction composites for simultaneous degradation of organics

**DOI:** 10.1038/s41598-022-05981-7

**Published:** 2022-02-09

**Authors:** Hesham Hamad, Mohamed M. Elsenety, Wagih Sadik, Abdel-Ghaffar El-Demerdash, Adel Nashed, Amr Mostafa, Shaimaa Elyamny

**Affiliations:** 1 Fabrication Technology Research Department, Advanced Technology and New Materials Research Institute (ATNMRI), City of Scientific Research and Technological Applications (SRTA-City), New Borg El-Arab City, Alexandria 21934 Egypt; 2 Department of Chemistry, Faculty of Science, Al-Azhar University, P.O. 11823, Nasr City, Cairo Egypt; 3 Materials Science Department, Institute of Graduate Studies and Research (IGSR), Alexandria University, Alexandria, Egypt; 4 Electronic Materials Research Department, Advanced Technology and New Materials Research Institute, City of Scientific Research and Technological Applications (SRTA-City), P.O. Box 21934, New Borg El-Arab City, Alexandria Egypt

**Keywords:** Catalysis, Environmental chemistry, Inorganic chemistry, Materials chemistry, Physical chemistry, Surface chemistry, Theoretical chemistry

## Abstract

The necessity to resolve the issue of rapid charge carrier recombination for boosting photocatalytic performance is a vigorous and challenging research field. To address this, the construction of a binary system of step-scheme (S-scheme) CuO@TiO_2_ heterostructure composite has been demonstrated through a facile solid-state route. The remarkably enhanced photocatalytic performance of CuO@TiO_2_, compared with single TiO_2_, which can consequence in the more efficient separation of photoinduced charge carriers, reduced the band gap of TiO_2_, improved the electrical transport performance, and improved the lifetimes, thus donating it with the much more powerful oxidation and reduction capability. A photocatalytic mechanism was proposed to explain the boosted photocatalytic performance of CuO@TiO_2_ on a complete analysis of physicochemical, DFT calculations, and electrochemical properties. In addition, this work focused on the investigation of the stability and recyclability of CuO@TiO_2_ in terms of efficiency and its physical origin using XRD, BET, and XPS. It is found that the removal efficiency diminishes 4.5% upon five recycling runs. The current study not only promoted our knowledge of the binary system of S-scheme CuO@TiO_2_ heterojunction composite photocatalyst but also shed new light on the design of heterostructure photocatalysts with high-performance and high stability.

## Introduction

Recently, the developments of demand for fabrication of new semiconductors with various characteristics have been grown by the dynamic progress of the chemical industry. TiO_2_ has gotten a lot of interest in photocatalysis because of its strong oxidizing capability, long-term photostability and lack of toxicity^1,2^. However, the traditional photocatalysis using TiO_2_ is restricted by various factors including the fast recombination of photogenerated electron (e^−^) and holes (h^+^), low absorption of solar light, and low photocatalytic activity on the conventional crystal facets, which meaningfully hinders the practical application till now, this problem is unsolved^3,4^. So, the formation of heterojunction donates the highly efficient separation of photo-generated e^−^/h^+^ pairs by modification of TiO_2_ surface with other semiconductors can be an active strategy to boost the efficiency of photocatalysis process^5^.

Copper oxides have a narrow band gap and are a p-type semiconductor. Cupric oxide (CuO) and cuprous oxide (Cu_2_O) are the two types of crystals of copper oxides^6^. CuO is a monoclinic phase, whereas Cu_2_O has a cubic structure, which CuO is more thermostable than Cu_2_O^7^. As a sensitizer of TiO_2_, CuO is an appealing substance. As a result, the heterojunction between n-TiO_**2**_ and p-CuO is mainly promising in this regard, as it enables the separation and transit of photo-induced charge carriers in CuO@TiO_2_, and subsequently minimizes the possibility of recombination and increases the photocatalytic capabilities^8^. Due to the formation of CuO–TiO_2_ p–n heterojunction, the photo generated holes migrate towards the interface, whereas the electrons migrate towards the bulk. As a result, the p–n junction is predictable in terms of charge carrier production and lifetime, which has a positive impact on the photocatalytic performance^9,10^.

Recently, a step (S)-scheme heterojunction photocatalysts with spatially separated reduction and oxidation units is hot topic because the photoinduced electrons and holes accumulate in the semiconductor with the more negative conduction band (CB) position and the other semiconductor with the more positive valence band (VB) position, respectively, in the recently. Such a novel heterojunction ensures visible light absorption, enhanced charge separation, and higher charge carrier redox capacity all at the same time. Aside from the two semiconductors' matching band structures, an intimate interfacial contact between them is critical for promoting the S-scheme charge transfer path across the interface^11,12^.

Based on the above research background and assumptions, to date, no study has been focused on the photocatalytic application of a binary system of S-scheme CuO@TiO_2_ heterojunction nanocomposite. Herein, the goal of this research was the demonstration of facile solid-state synthesis of a binary system of S-scheme CuO@TiO_2_ heterojunction nanocomposite which was built by surface modification of TiO_2_ by CuO. The heterojunction formed between TiO_2_ and CuO induces the efficient separation of photogenerated e^−^/h^+^. We found that the surface modification strongly influences the properties of the produced photocatalyst (structure, composition, etc.) that are characterized by various physicochemical techniques and correlated with the photocatalytic performance of the samples. The enhancement of photocatalytic activity was proposed and explained in detail according to the mechanism of e^−^/h^+^ separation. In this inquiry, two crucial properties of CuO@TiO_2_, namely stability, and reusability, were also examined, particularly in terms of industrial-scale application.

## Results and discussions

### Structural analysis

**Figure 1:**
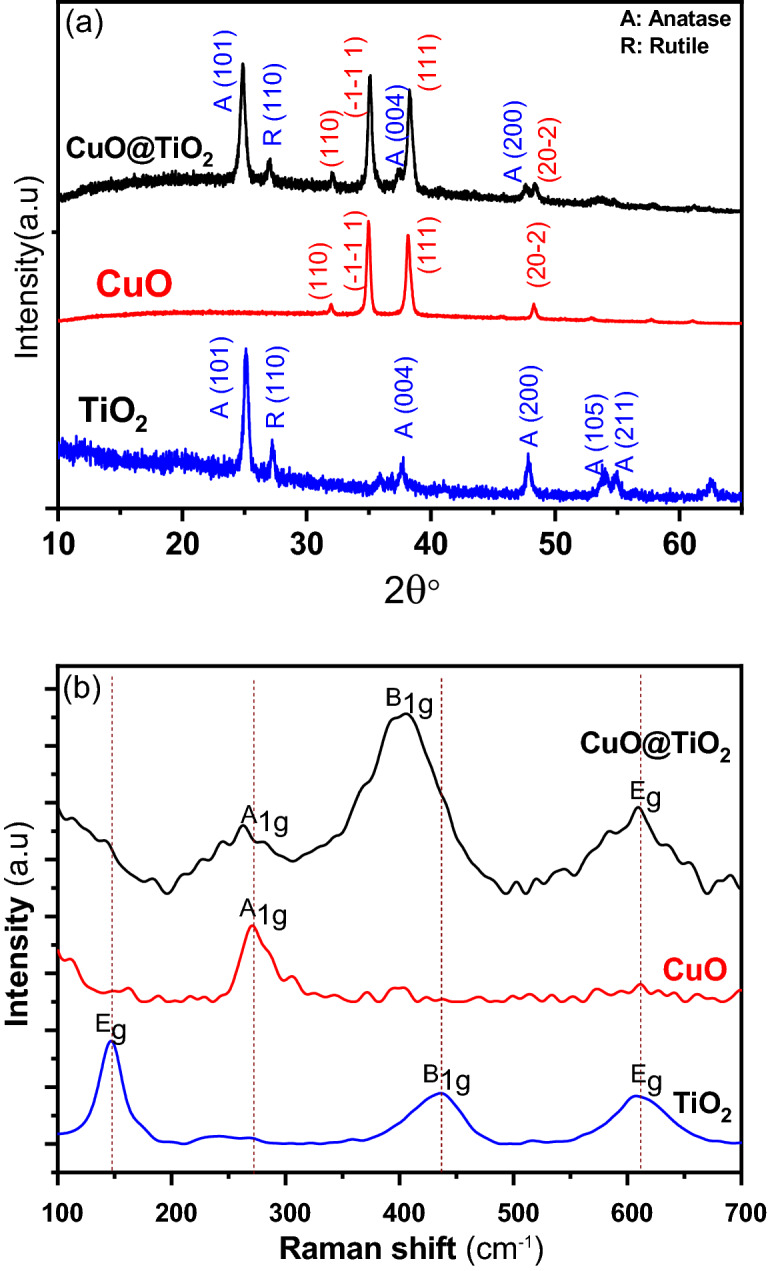
(**a**) XRD and (**b**) Raman spectra of TiO_2_, CuO, and its binary CuO@TiO_2_ heterojunction nanocomposites.

The structural characteristics of pure CuO and pure TiO_2_ powder were characterized as the contrasts to binary CuO@TiO_2_ heterostructure nanocomposites that shown in Fig. 1a. The diffraction peaks at scattering angles (2θ) of 32.0, 35.95°, 38.21° and 48.34° were indexed to (1 1 0) (− 1 − 1 1),(1 1 1), and (2 0 2) planes of monoclinic structure of CuO, respectively, which corresponding to JCPDS No. 48-1548^13^. Furthermore, the P-25 consists of anatase and rutile phases, matching very well with the confirmation of JCPDS No. 021-1272 and 021-1276, respectively^14^. Pure TiO_2_ and CuO peaks exhibit the high intensity and sharp nature, indicating pure crystalline nature. Other phases, such as impurities, Cu_2_O or metallic Cu, are not present, indicating that pure CuO could be achieved by chemical and calcination methods. The findings show that the binary composite of CuO@TiO_2_ heterostructures contains two-phase TiO_2_ and CuO compositions after the solid state reaction. CuO@TiO_2_ heterostructure nanocomposites show more XRD peaks than TiO_2_, indicating that CuO is present on the surface of the TiO_2_^15^. The higher intensity of (− 1 − 1 1) and (111) diffraction peaks in CuO@TiO_2_, could be due to the sheild effect from the CuO nanoparticles^16^. However, a small gradual shift of the characteristic peak (101) of TiO_2_ is observed towards a lower 2θ value after the formation of CuO@TiO_2_, which could be attributed to partial substitution of titanium atom (ionic radius Ti^4+^, 0.074 nm) by the larger size of Cu atom (ionic radius Cu^2+^_,_ 0.087 nm) at surface interaction^17,18^, as we suggested and studied by DFT calculations. The same phenomena were studied and confirmed due substitution of I atom by another halogen of Br and Cl atoms in lead-free perovskites^19^.

Moreover, it is vital to note that Raman spectroscopy is a surface-probing technique, whereas XRD samples analyze the bulk. As a result, Raman spectra revealed a surface modification caused by the vibrational mode. Figure 1b illustrates the Raman spectra of TiO_2_, CuO and its binary composite CuO@TiO_2_ heterostructure. In the Raman spectrum of TiO_2_, the active vibrational modes are recognized as: (i) the symmetric vibration of O–Ti–O in TiO_2_ due to the doubly E_g_ modes at 145 and 606 cm^−1^; and (ii) the symmetric bending vibration of O–Ti–O due to the B_1g_ modes at 437 cm^−1^, while the A_g_ peak at 272 cm^−1^ mode of CuO crystals, endorses the anatase-TiO_2_ phase's distinctive peaks as reported in the literature^20,21^. After solid state reaction, the modification by CuO affected on the position of the Raman peaks associated with the B_1g_ and A_1g_ vibration modes and confirmed the structural phases of TiO_2_ and CuO for binary CuO@TiO_2_ heterostructure as shown in Fig. 1b. Additionally, there is a blue shift at 404 cm^−1^ which established the robust interaction obtainable in CuO@TiO_2_. Cu^2+^ has a lower valence than Ti^4+^, therefore oxygen vacancies and other crystalline defects generated at the surface of TiO_2_ by CuO helped to explain the spectrum behavior^22,23^. The structural and vibrational features of the TiO_2_ lattice were altered when CuO was added, demonstrating that CuO and TiO_2_ had a strong interaction between them^24^. Surface defects like this could act as photoactive centers, improving the charge separation efficiency^23^.

### Microstructure and elemental distribution

**Figure 2:**
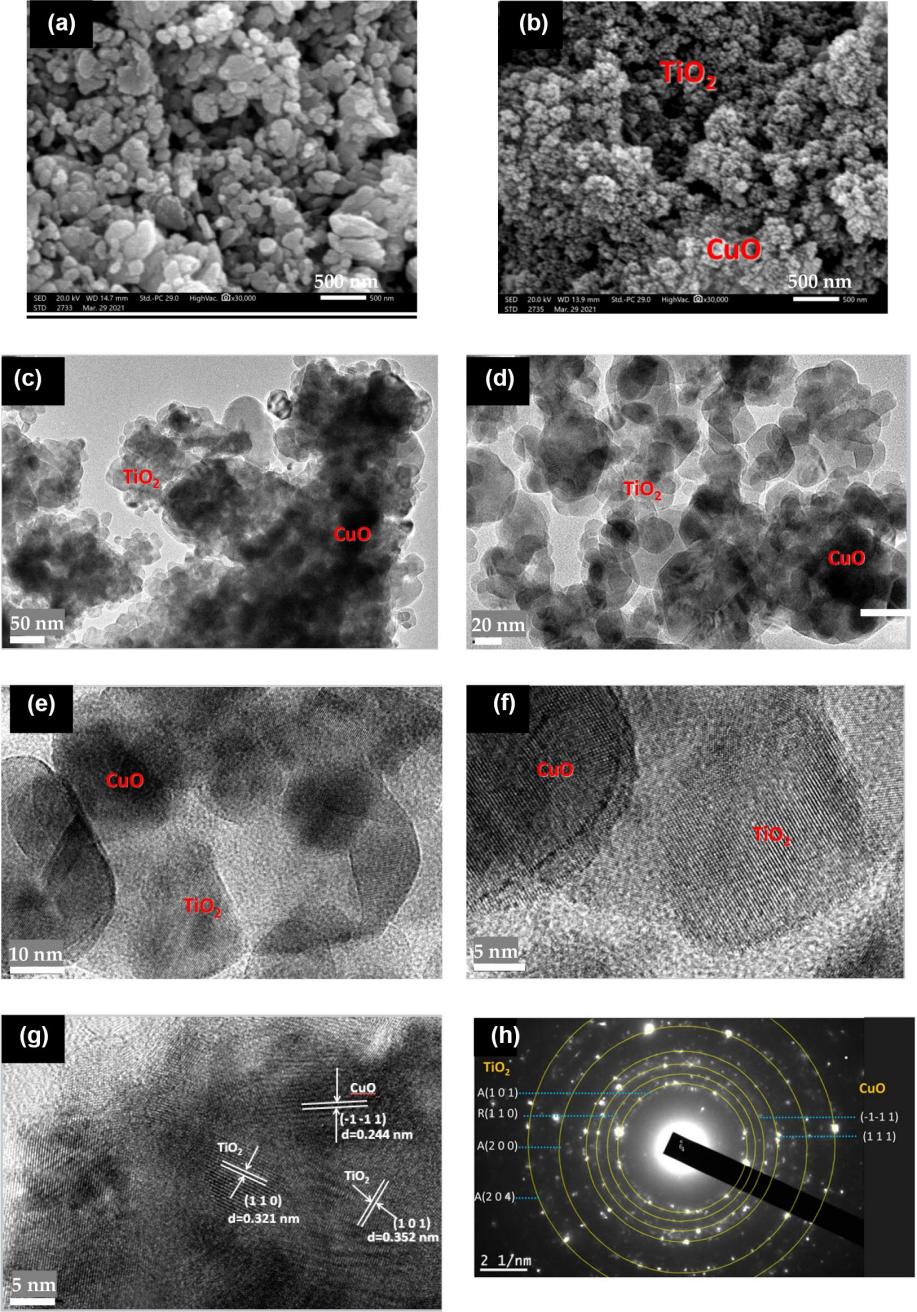
SEM images of (**a**) CuO, and (**b**) CuO@TiO_2_, (**c**–**g**) HR-TEM images of CuO@TiO_2_, and (**h**) SAED of CuO@TiO_2._

The microstructure of CuO and its binary system of CuO@TiO_2_ heterostructure using SEM is shown in Fig. 2. Figure 2a provides an overview of the morphology of the CuO NPs which exhibit aggregated nanoparticles associated with irregular shape. In Fig. 2b, after solid state reaction, it revealed that the aggregated and interlinked nanocrystals, that aid in the intraparticle charge transfer. CuO nanoparticles also existed on the surface of TiO_2_ nanoparticles.

The detailed morphology and microstructure of binary CuO@TiO_2_ heterostructure nanocomposite were further investigated by high resolution transmission electron microscope (HR-TEM) and energy-dispersive X-ray spectroscopy (EDS). The corresponding HR-TEM image of CuO@TiO_2_ heterostructure with different magnifications is presented at Fig. 2c–g. It is found that the extremely aggregated with irregularly shaped nanoparticles. The light gray regions are the TiO_2_ microstructure, and the dark regions are CuO nanoparticles. This proved that the binary system of CuO@TiO_2_ nanocomposite was crystalline in a uniform shape (not a physical mixture), but had the decoration of CuO on TiO_2_ which formed the interface that leads to the CuO@TiO_2_ heterojunction. Furthermore, it is well understood that having a large number of homogenous nanopores is favorable because it allows for the quick transfer of light-excited carriers to the particle surface, substantially lowering the carrier recombination rate and speeding up the photocatalytic reactions^25^. The d-spacing value of TiO_2_ was for the (101) and (110) crystal facets of TiO_2_ had d-spacing values of 3.52 and 3.22 Å, respectively, whereas and the (− 110) crystal facets of CuO had d-spacing value of CuO was 2.45 Å (Fig. 2g). The heterojunction of CuO@TiO_2_ is further confirmed by these findings^26^. The selected area electron diffraction (SAED) pattern in Fig. 2h presented the consistency with the monoclinic CuO {− 1 − 1 1}, and {1 1 1} and P25 TiO_2_ for anatase {1 0 1}, {2 0 0},and {2 0 4} planes and rutile {1 1 0}and revealed its polycrystallinity demonstrating that both the planes are present as CuO@TiO_2_ heterojunctions (Fig. 2h).

**Figure 3:**
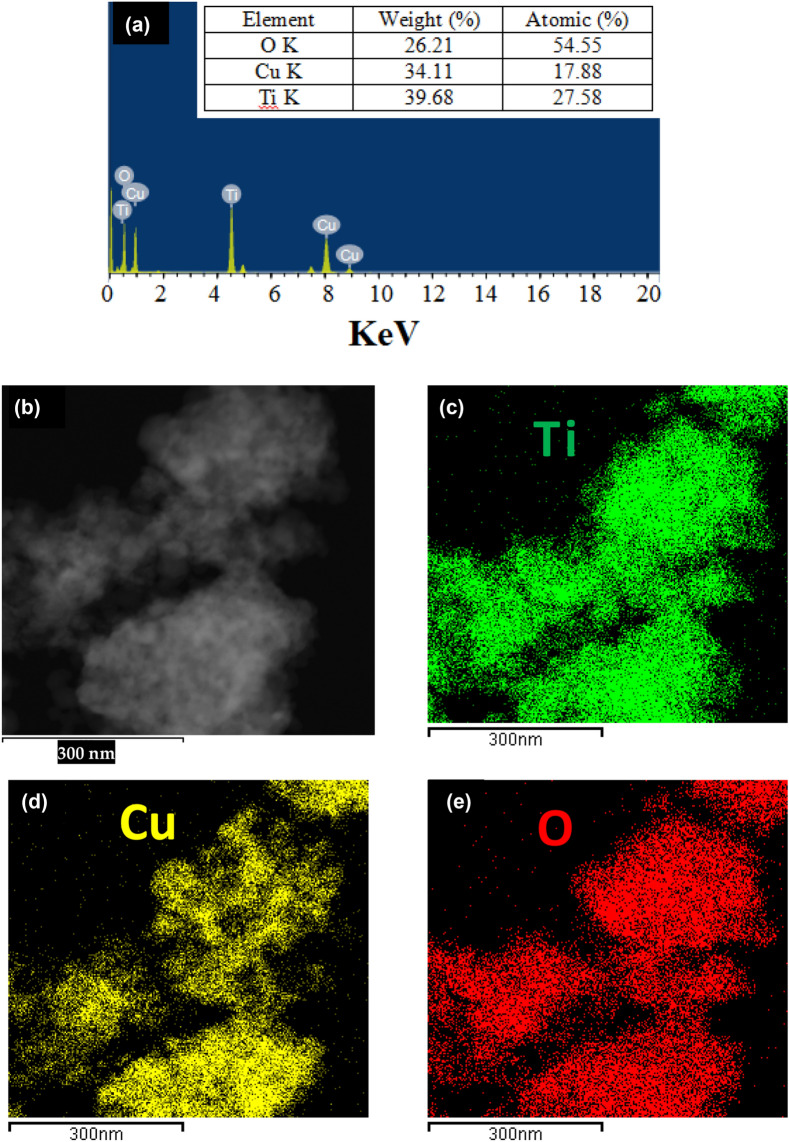
(**a**) EDS, (**b**) TEM image, and (**c**–**e**) corresponding elemental mapping of binary system of CuO@TiO_2_ heterostructure nanocomposites: (**c**) Ti, (**d**) Cu, and (**e**) O.

The elemental analysis in the binary system of CuO@TiO_2_ nanocomposite were studied by energy-dispersive X-ray spectroscopy (EDS) mapping Fig. 3a endorses the existence of copper (Cu), titanium (Ti), and oxygen (O) elements with the inset displaying an atomic percentage proportion. The EDS mappings of each element are shown in Fig. 3b–e, and they clearly show that this Cu, Ti, and O species are homogeneously distributed throughout the entire selected area, demonstrating the continuous existence of O in the CuO particle and TiO_2_ and further through their interface (Fig. 2e). This clearly illustrates the construction of the heterojunction between CuO and TiO_2_, which assists in the transfer of charges between the bands of two semiconductors, CuO and TiO_2_^27^.

### Chemical valence states

**Figure 4:**
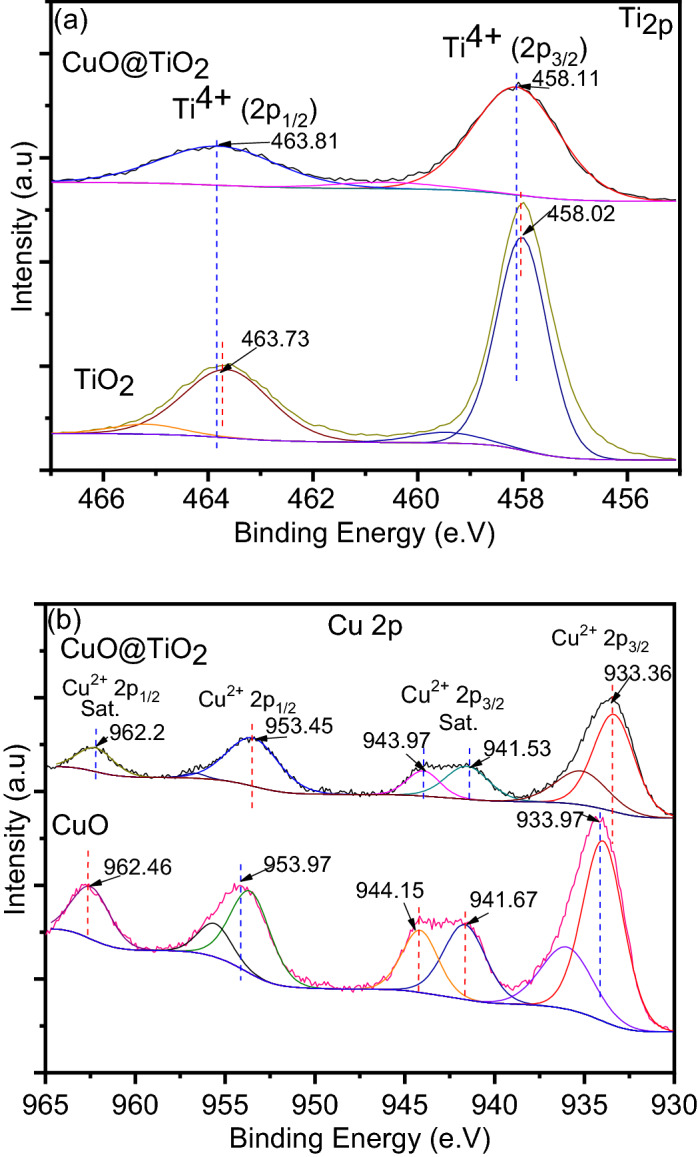
(**a**) High-resolution XPS scan of (**a**) Ti 2p, and (**b**) Cu 2p.

The chemical surface of pure TiO_2_, pure CuO, and its binary system CuO@TiO_2_ heterostructure was analyzed by XPS. The high resolution of XPS spectrum of Ti, O, and Cu is displayed in Fig. 4. The binding energy values can be utilized to infer information about Ti chemical and electronic structure states in TiO_2_^28^. As shown in Fig. 4a, the Ti 2p_3/2_ and Ti 2p_1/2_ peaks of Ti^4+^ ions in the lattice oxide have binding energies of 458.11 and 463.81 eV, respectively, for CuO@TiO_2_. The Ti 2p_2/3_ and Ti 2p_1/2_ binding energies differ by 5.7 e.V, indicating that Ti is mainly Ti^4+^^28^. When compared to the binding energies of TiO_2_, the small positive shifts of both Ti 2p_2/3_ and Ti 2p_1/2_ are meaningful of two distinct TiO_2_ entities and suggest a variation in the electronic state of Ti in Ti–O^23,29^. Also, no additional peaks may be observed on the spectra of CuO@TiO_2_ in the Ti_2p_ region, which indicates that the addition of CuO is not disturbing the TiO_2_ lattice and both oxides exist as separate phases in presented materials^30^. It also confirms CuO to TiO_2_ electron transport at CuO@TiO_2_ heterojunction^31^. These results are supported by HRTEM. The only observed change in the Ti 2p XPS image is the decrease of intensity of the peaks, which is the natural consequence of the decreasing concentration of the TiO_2_ in the materials with the addition of CuO.

Based on these assignations, the O_1s_ region on the spectra of TiO_2_, CuO, and its binary system shows three peaks after deconvolutions, which resolved to three types of oxygen atoms, the lattice oxygen (O_L_), surface hydroxyl groups (O_H_), and surface oxygen vacancies (O_V_) (Supplementary Fig. S1). The position of O_L_ is 529.44 and 529.46 eV for TiO_2_ and CuO, respectively. The property of O_L_ was ascribed to the lattice O^2−^ anions bonding to the metal cations in the Ti–O or Cu–O, has the lowest binding energy of O_1s_ at 529.39 eV for CuO@TiO_2_^32^. The broad shoulder at 530.53 eV was linked to the surface hydroxyl groups (O_H_) on the surface^27^, while at peak at 530.94 eV refers to the surface oxygen vacancies (O_V_)^33^. According to the calculation of peak areas, the concentration of O_V_ is 35.87% over CuO@TiO_2_ is higher than CuO (22.89%) and TiO_2_ (13.32%), which is crucial for photocatalytic activity. The chemical composition of various types of oxygen and their percentages are summarized in Table 1.

**Table 1:** Species percentage and corresponding binding energies (in brackets, eV) were obtained for TiO_2_, CuO, and its binary system CuO@TiO_2_.

Sample	O_L_ (%)	O_H_ (%)	O_V_ (%)
TiO_2_	71.07 (529.44)	15.56 (530.81)	13.37 (528.86)
CuO	51.1 (529.46)	26.10 (531.01)	22.89 (532.34)
CuO@TiO_2_	24.36 (529.39)	39.77 (530.53)	35.87 (530.94)

From Fig. 4b, there is two prominent peaks at 933.36 and 953.45 eV can be endorsed to Cu 2p_3/2_ and Cu 2p_1/2_, compared to 933.97 and 953.97 eV for CuO, respectively^23^. Also, two addition peaks at 942.66 and 962.2 eV can be assigned to the satellite peaks for Cu^2+^, compared to 941.67 and 962.46 for CuO, respectively. No Cu _2p_ peaks shift and no additional peaks are forming when TiO_2_ concentration is increasing in the CuO. The formation of Cu^2+^ species rather than Cu^+^ in the composite is confirmed by B.E gaps of 20.09 eV^16^. The higher binding energy comes from the upshift of Cu_2p_ peak that shows the substantial interaction between CuO nanoparticles and TiO_2_ nanoparticles, which is consistent with the literature^34,35^. Furthermore, there is no peak that relates to Cu^+^ at around 932.7 eV, signifying that Cu species mainly exist as CuO. The existence of an unfilled Cu 3d shell corresponds to the Cu^2+^ species at the CuO surface, as seen by the shakeup satellite peaks with binding energy at 942.66 and 962.2 eV^36^. This is another proof to endorse the dominant surface copper is CuO in CuO@TiO_2_ heterostructure.

In summary, the surface of CuO@TiO_2_ photocatalyst is oxidized and functional groups such as OH are incorporated during the calcination. The oxygen vacancies in CuO@TiO_2_ form free electrons that lead to creating a reduced form of Ti species (e.g. Ti^4+^). This is endorsed by the blue shift of the B_1g_ vibrational mode of TiO_2_ (as identified by Raman) and the negative shift of Ti 2p to lower binding energy (as identified by XPS). Based on the XRD, HR-TEM, XPS, and Raman spectroscopy observations, we endorse the existence of TiO_2_ and CuO and the successful fabrication of CuO@TiO_2_ heterostructure photocatalysts with strong interface interaction.

### Textural properties

**Figure 5:**
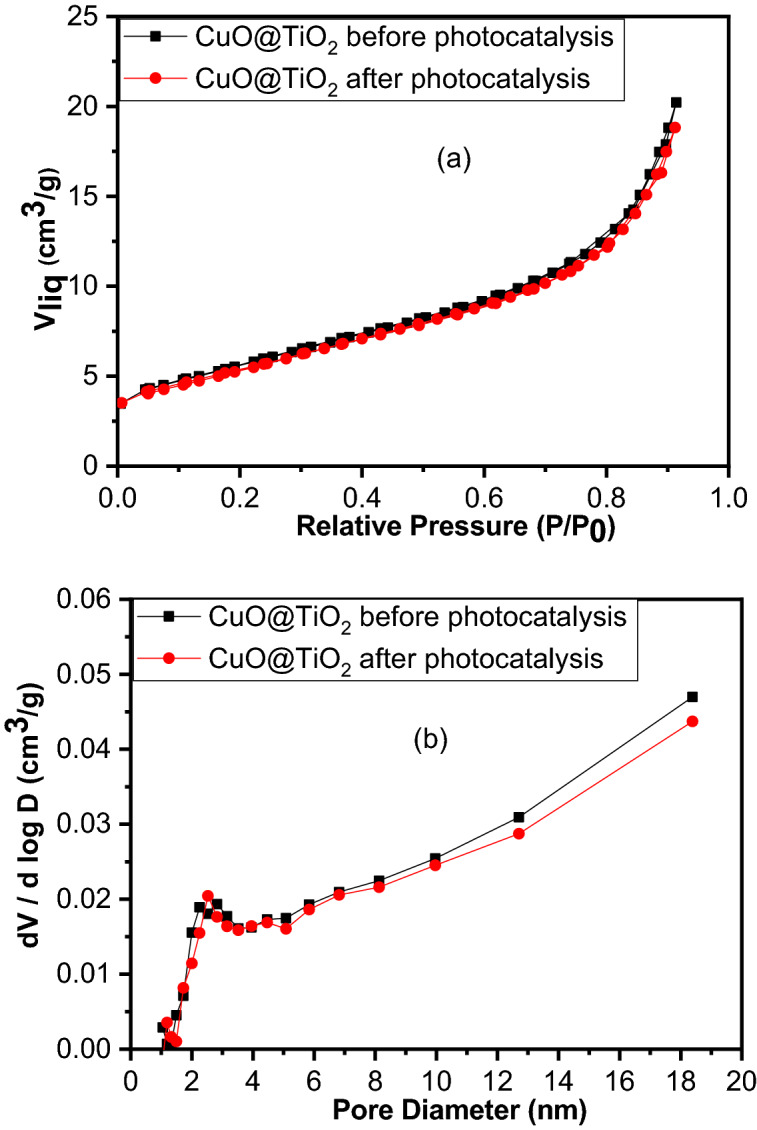
(**a**) N_2_ adsorption/desorption isotherms, and (**b**) pore diameter distributions of CuO@TiO_2_ heterostructure before and after photocatalysis.

The textural nature of the catalyst CuO@TiO_2_ investigated using BET analysis. The results showed that it has a surface area of 19 m^2^/g, a total pore volume of 3.12 × 10^–2^ cm^3^/g, and a pore diameter of 6.08 nm. Based on the IUPAC classification, the nitrogen sorption isotherms for the catalyst display the type IV in the shape with H3 hysteresis loop and large adsorption of N_2_ at P/P_0_ > 0.8 (Fig. 5a). H3 loop indicates the existence of large mesopores (2–50 nm) with few of micropores^37^. Figure 5b depicts the plot of the BJH pore-size distribution of the catalyst CuO@TiO_2_, which shows that it is essentially mesoporous particles^38^. The low surface area of CuO@TiO_2_ confirms the well-formed crystalline structure, which is extremely important for photocatalytic applications. The high pore volume of the CuO@TiO_2_ increases the internal mass transfer and therefore improves the catalytic activity and enhances the photodegradation of AR8.

### Optical properties

**Figure 6:**
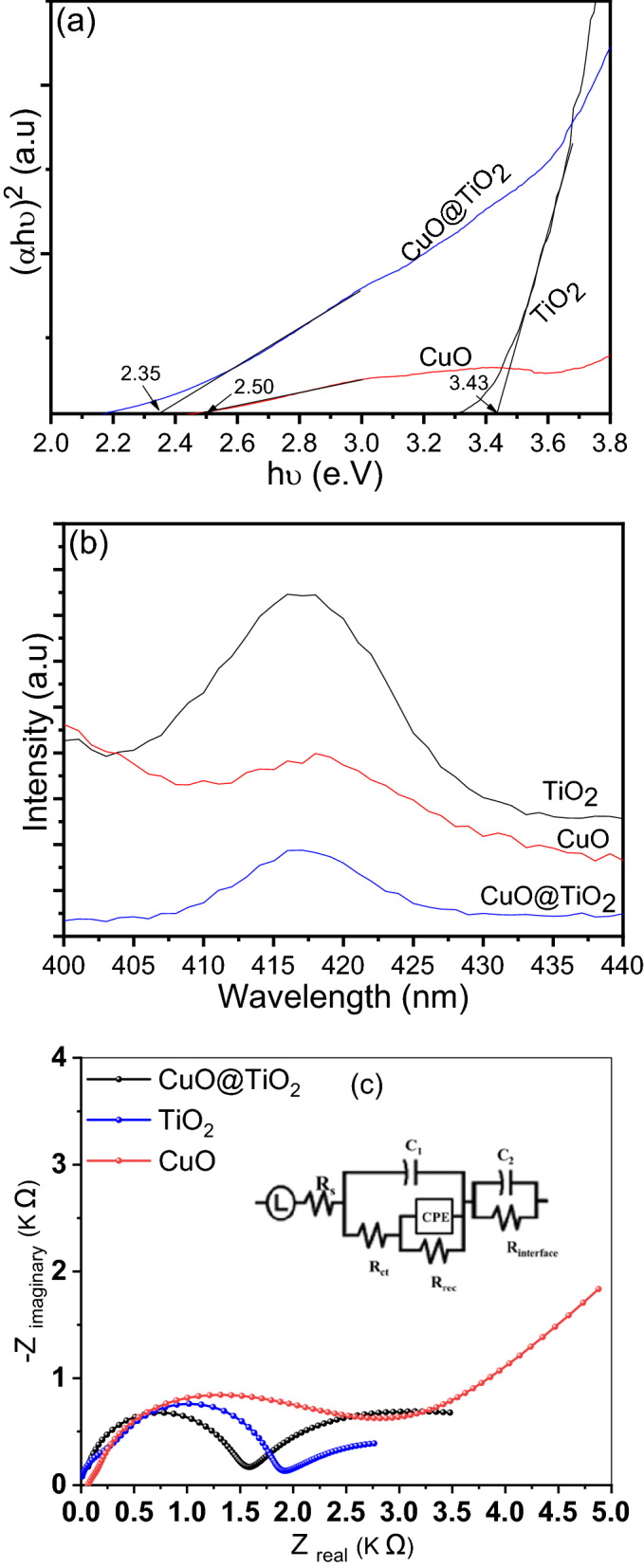
(**a**) Tauc plots of the band gap, (**b**) Photoluminescence spectra, and (**c**) EIS profile of photocatalysts.

The band gap of TiO_2_, CuO, and its binary CuO@TiO_2_ heterostructure catalysts by using the tauc plot are shown in Fig. 6a. It represents the direct transition of band gap energy by plotting (αhυ)^2^ versus hυ. As seen, the band gap for P-25 TiO_2_ was correspondingly to 3.43 eV, which is caused by the intrinsic interband absorption of TiO_2_^39^. After sintering at high temperature, the Cu_2_O color was changed to black, matching the color of the CuO semiconductor. After addition of CuO nanoparticles distinctively displays the lower optical band gap energy of 2.35 eV compared to CuO and TiO_2_ of about 2.5 and 3.43 eV respectively. The smaller bandgap of CuO@TiO_2_ heterostructure as compared to TiO_2_ can be elucidated on the basis of (a) the quantum confinement phenomena, and (b) the existence of CuO favors the formation of oxygen vacancies and/or the partial reduction of Ti sites (as identified by XPS). Taking into account the reduction of Ti sites and/or oxygen vacancies acts as a new state localized in the decreasing of the bandgap. The incorporation of CuO in TiO_2_ matrix results in the formation of two closely spaced conduction bands formed by the sharing of interfacial electrons and holes between Cu^2+^ and Ti^4+^. Therefore, the enhanced light absorption and interfacial charge transfer will be advantageous for improving the photocatalytic performance of the hybrid photocatalysts^21,40^ As a result, the latter optical adsorption is extra proof for the presence of CuO classes in the CuO@TiO_2_ heterostructure.

To determine the role of photogenerated electron–hole pairs in TiO_2_, CuO, and its binary CuO@TiO_2_ heterojunction, as well as to demonstrate the mechanism liable for the remarkable boosted photocatalytic degradation of pollutants. Photoluminescence (PL) studies were investigated. The fluorescence emission spectra of samples exhibited a broadband peak at 423 nm under the excitation of 350 nm (Fig. 6b). As we know, the fluorescence intensity of the catalysts tended to be inversely proportional to the rate of recombination rate of e^−^/h^+^^41^. As a result, the fluorescence intensity of the pure TiO_2_ and CuO is high, indicating a higher photogenerated electron–hole binding rate^41^. The modification of TiO_2_ by CuO was found to lower fluorescence intensity and more efficient separation of electron and hole by CuO@TiO_2_ heterojunction that formed at the interface and therefore accounts for boosting the photocatalytic activity^42^. It also accelerates the creation of e^−^, and extends the lifetime of e^−^/h^+^ lifetime, which was ascribed to the potential well made by the Cu^2+^ hetero-junction to trap electrons^43^. The separation of electrons and holes results from the electron transfer from TiO_2_ to CuO nanoparticles at the interface of CuO (p-type) and the electron-rich of TiO_2_ (n-type), which is one possible clarification for the suppressed charge recombination^44,45^.

### Electrochemical properties, EIS

To acquire profoundly thoughtful into the influence of CuO nanoparticles on behaviors of charge transport and reaction rate of the surface of binary system of CuO@TiO_2_ composite, the estimation of electrochemical impedance spectroscopy (EIS) was achieved. It is well acknowledged that the smaller arc radius of the first semi circuit matches to a reduced electron-transfer resistance (the low-frequency semicircle), implying the high efficiency of separation and charge transfer^46^, however second semi circuit is attributed to recombination resistance (the high-frequency semicircle) in p–n junction of photovoltaic devices in the fitting EIS spectra (Fig. 6c). The semicircular in EIS plots for all samples indicate the same behavior. Furthermore, the equivalent electrical circuit was shown as inset and the collected values of ohmic series resistance (R_s_), charge transfer resistance (R_ch_), recombination resistance (R_rec_), and interface resistance were detailed in Table 2. Among all results the CuO@TiO_2_ show a significantly smaller charge transfer resistance R_ct_ (1372 Ω) than both of TiO_2_ (1476 Ω) or CuO (3074 Ω), which suggests that CuO@TiO_2_ has the fastest electron–transfer rate^47,48^. Comparing with bare CuO and bare TiO_2_, the binary system of CuO@TiO_2_ suggests the higher separation efficiency of photoinduced e^−^/h^+^ pairs at the interface, faster interfacial charge transfer, and more efficient separation of electron–hole pairs^46,49^. On the other hand, the CuO@TiO_2_ shows higher recombination resistance than TiO_2_ which confirms that the additional CuO interface of the binary system of CuO@TiO_2_ prevents the excited electrons from recombination in TiO_2_. It is worth to mention, that the equivalent electrical circuit present also very high interface resistance for both TiO_2_ (315 Ω) and CuO (1383 Ω) compared to the binary system CuO@TiO_2_ (4 Ω). Therefore, the lower charge transfers resistance and higher recombination resistance of CuO@TiO_2_ favored a higher photocatalytic activity.

**Table 2:** Ohmic resistance values obtained from the EIS analysis of TiO_2_, CuO, and its binary system of CuO@TiO_2_ heterojunction. (Where: R_s_ (series resistance), R_ch_ (charge transfer resistance), R_rec_ (recombination resistance).

Photocatalyst	R_s_ (ohm)	R_ct_ (ohm)	R_rec_ (ohm)	R_interface_ (ohm)	X^2^
TiO_2_	0.1	1476	1992	315	0.01
CuO	21	3074	1.6E15	1382	0.01
CuO@TiO_2_	46	1372	3391	4	0.001

### Computational calculations

**Figure 7:**
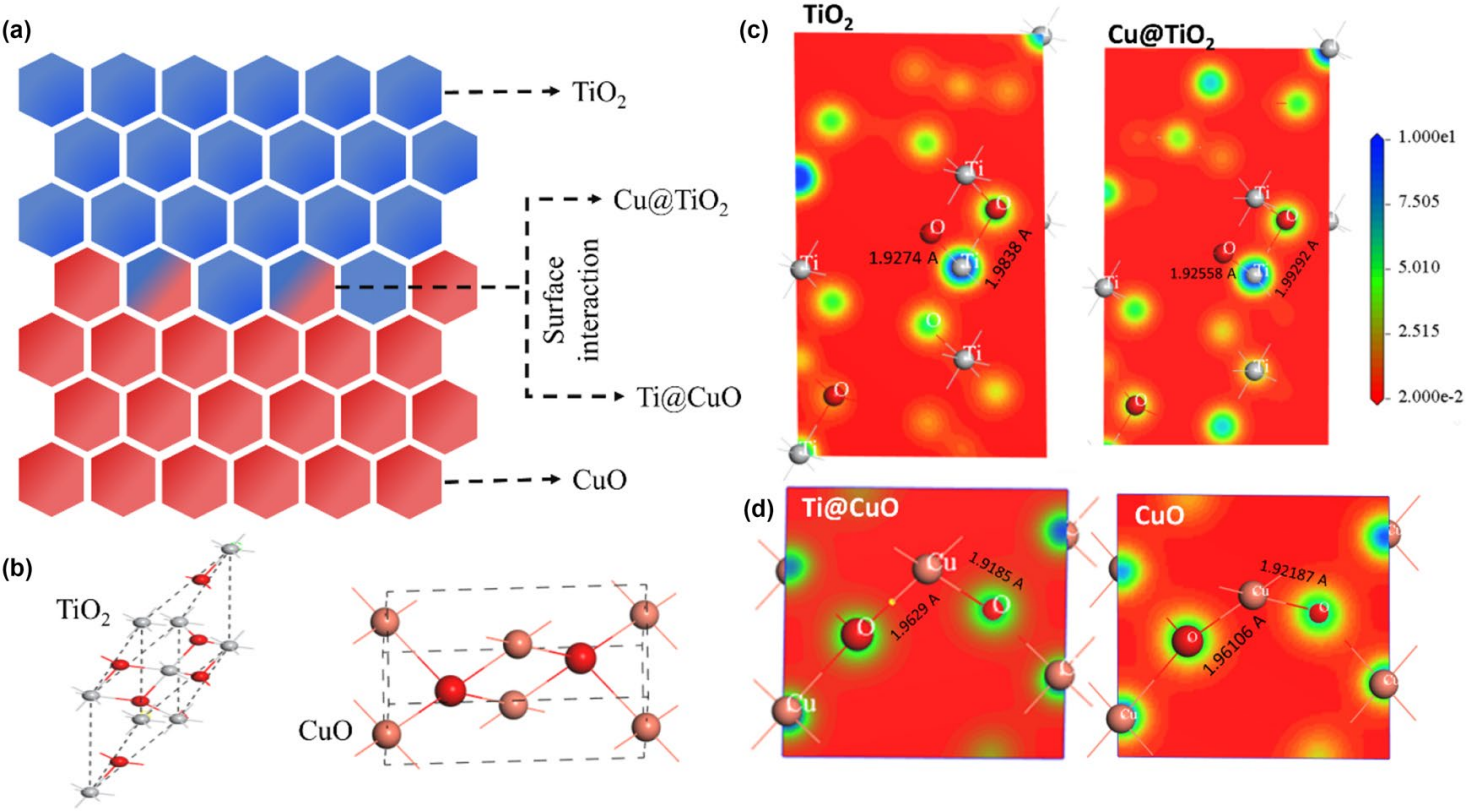
(**a**) Simulated surface interaction of (**a**) CuO and TiO_2_; Crystal structure unite cell of (**b**) both TiO_2_ and CuO; charge density distribution at the best fit plan for both (**c**) TiO_2_, (**d**) CuO and two proposed surface interaction Cu@TiO_2_ and Ti@CuO as well (Cu–O and Ti–O bond length by angstrom Å).

The DFT calculations are commonly regarded as crucial to comprehending the behavior of chemical structures. We presented four possible configurations based on the XRD results (TiO_2_, Cu@TiO_2_, Ti@CuO, and CuO) that might exist as illustrated in Fig. 7. The computational calculations were performed to relax all possible structures and estimate the charge distribution and electronic properties as well^50–52^. Along best-fit plane's charge density distributions for the four possible configurations were carried out. The resulting data display a small displacement of Ti/Cu positions along with the surface interaction of both materials and combined with an increase in a bond length of Ti–O and Cu–O for both Cu@TiO_2_, Ti@CuO, respectively.

**Figure 8:**
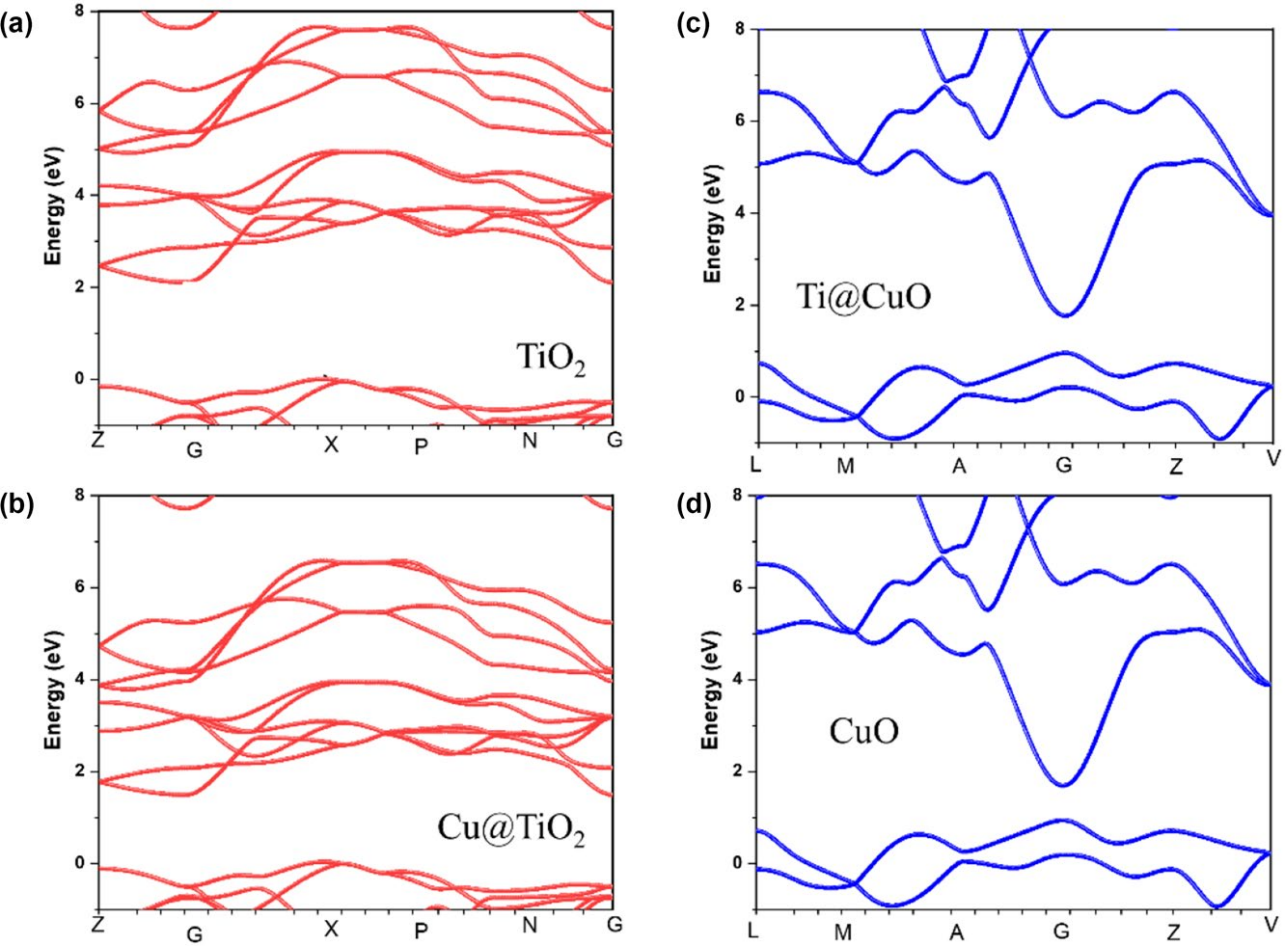
Bandstructure digram of four proposed configurations (**a**) TiO_2_; (**b**) Cu@TiO_2_; (**c**) Ti@CuO; and (**d**) CuO.

The experimental energy gap results of diffuse reflectance spectra are inconsistent with the preformed band structure calculations (Fig. 8). The band diagram shows an indirect bandgap for TiO_2_ and proposed Cu@TiO_2_ (surface interaction) from X to G symmetry point, along with the crystal symmetry directions in the first Brillouin zone. Similarly, the findings reveal a direct bandgap for both CuO and Ti@CuO at the G symmetry point. Meanwhile, the computed bandgap values show a gradual decrease with increasing Cu and Ti substitution ratio in both surface interaction configurations of Cu@TiO_2_ and Ti@CuO, respectively. Thus, the findings confirm the UV–Vis results and also predict enhancing of the performance of prepared catalysts toward photodegradation applications.

**Figure 9:**
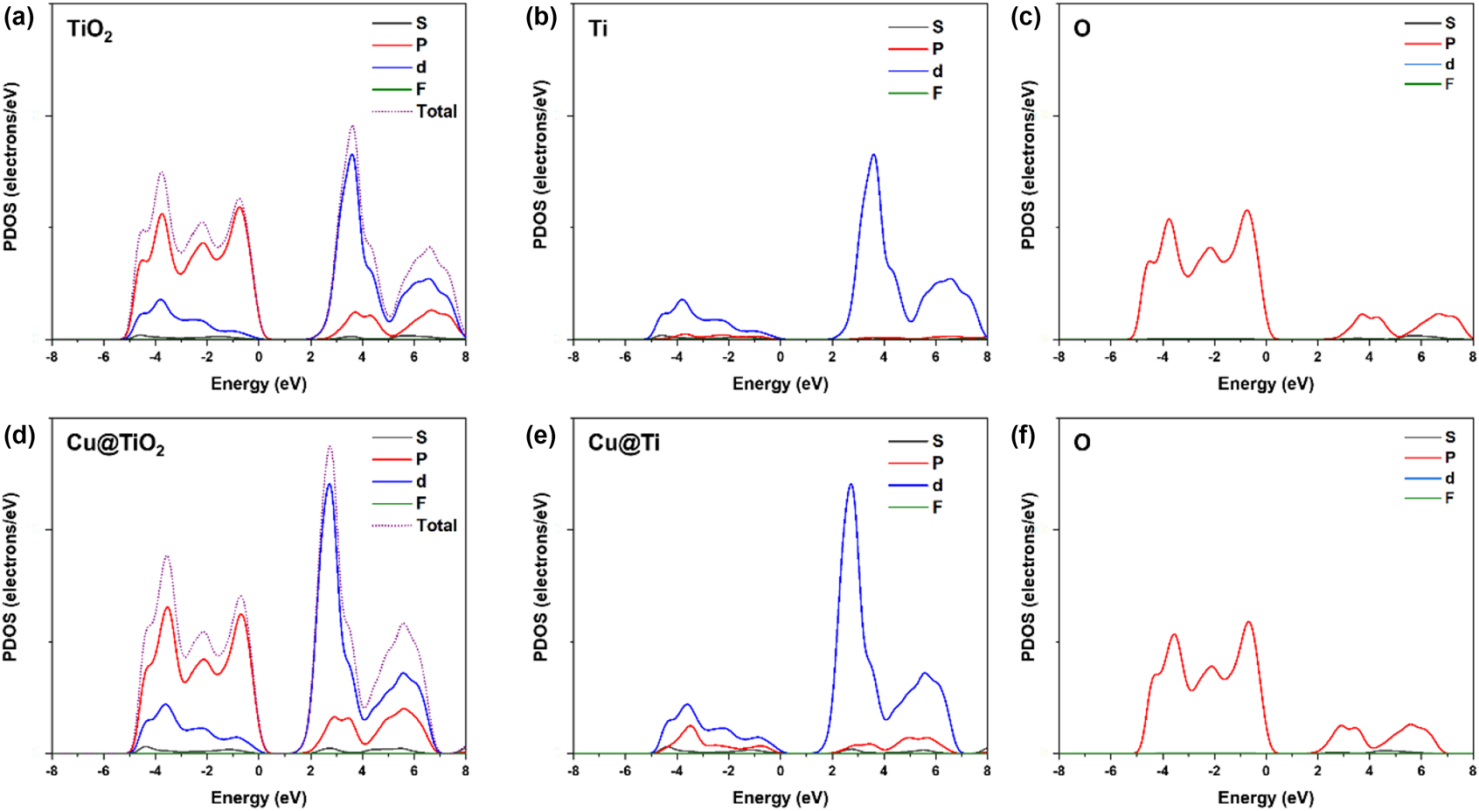
TDOS of (**a**) TiO_2_; PDOS of TiO_2_ based on (**b**) Ti and (**c**) O^−^ (**d**) TDOS of Cu@TiO_2_; PDOS of Cu@TiO_2_ based on (**e**) Cu/Ti^−^ and (**f**) O.

However, the TDOS result (Fig. 9) for the configurations of TiO_2,_ and Cu@TiO_2_ show a major contribution of antibonding *d-*orbital at the minimum CB and *p-*orbital at the maximum VB. For more clarifications, the partial density of state (PDOS) of Ti and O atoms of TiO_2_ indicates that the main contribution at maximum VB depends on the *p* orbital of O atoms, Meanwhile the *d-*orbital of Ti at the minimum CB. Similarly, Cu doped Cu@TiO_2_ refers to the contribution of the same orbital with a significant effect at minimum CB toward lower bandgap which clarifies the reason behind that.

**Figure 10:**
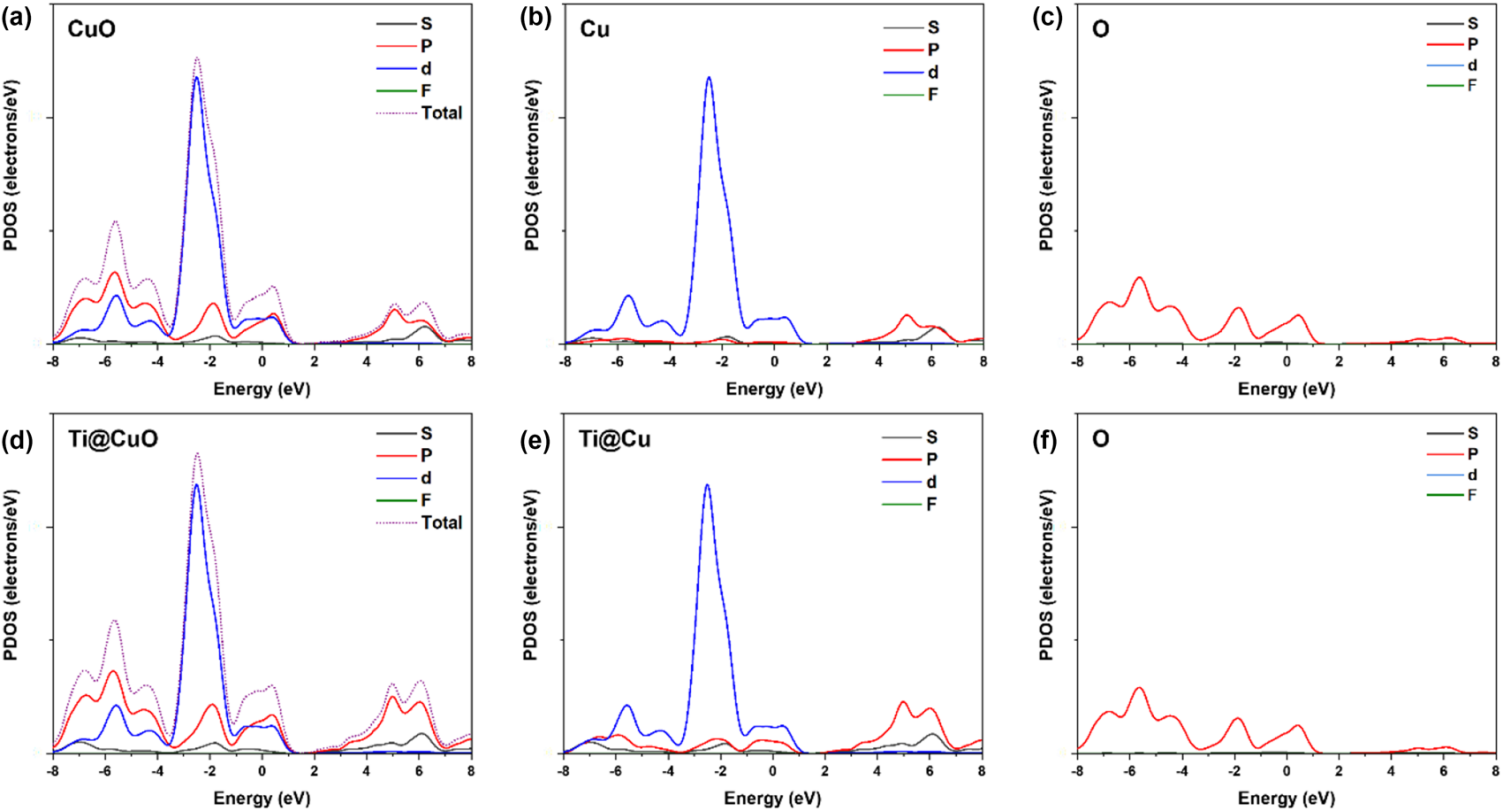
TDOS of (**a**) CuO; PDOS of CuO based on (**b**) Cu and (**c**) O. TDOS of (**d**) Ti@CuO, PDOS of Ti@CuO based on (**e**) Ti/Cu and (**f**) O.

The total density of state (TDOS) for CuO and Ti@CuO (Fig. 10) refer to a probability of (*p* and *d*) orbitals contributes in the VB and *d-*orbital at the minimum of the CB. However, PDOS of Oxygen (O), and Cupper (Cu) atoms of CuO show that at maximal VB, the main contribution is dependent on both *p*-orbital of O, and *d-*orbital of Cu atoms. Also, the *p-*orbital of Cu has a main contribution at the minimum conduction band without any sign of contribution of O atoms in conduction band. Likewise, Ti doped Ti@CuO at the surface interaction refer to the same orbitals with a significant contribution at maximum VB. Thus, these results at the surface interaction of CuO–TiO_2_ configuration predict that a significant enhancement of CB to decrease the band gap. However, because of the contribution of copper atoms to enhancement of maximum VB in the existence of the *p*-orbital of titanium atoms, could achieve the charge transfer due to S-scheme mechanism and resulting in an increase in the attract of excited electrons of CuO and the holes of TiO_2_ as well, during photocatalytic degradation process.

### Photocatalytic activity

#### Kinetic studies

**Figure 11:**
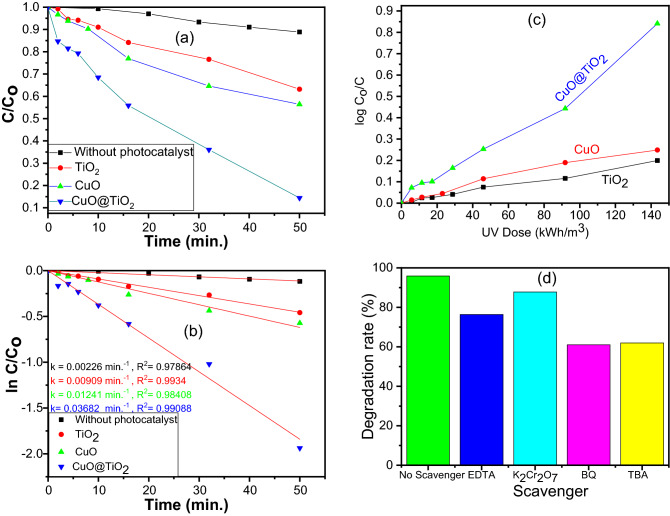
(**a**) The change in the concentration of Acid Red 8 dye as a function of exposure time (**b**) kinetic model of the Acid Red 8 photocatalytic degradation, (**c**) Change of log (C_o_/C) with UV dose for degradation of AR8 dye with different photocatalysts, and (**d**) Photocatalytic activity of AR8 dye over the CuO@TiO_2_ in the presence of different scavengers.

The photocatalytic degradation of Acid Red 8 (AR8) aqueous solution was assessed using batch mode. Figure 11a shows the catalytic activities of without catalyst (UV only), TiO_2_, CuO, and its binary CuO@TiO_2_ heterojunction composites. The photocatalytic activity of the CuO@TiO_2_was found to be substantially higher than those of TiO_2_ and CuO nanoparticles. The photocatalytic degradation of AR8 has also followed the pseudo-first-order kinetic model (Fig. 11b). Kinetic curves also highlight CuO@TiO_2_ exhibits 3.08 and 4.11 times the photodegradation ability of CuO and TiO_2_, respectively (Table 3). Also, the t_½_ decreases with increasing the apparent rate constant (k_app_) as shown in Table 3.

**Table 3:** Collective data of apparent rate constants, half lifetime, electrical energy per order, and apparent quantum yield for degradation of acid red 8 dye using TiO_2_, CuO, and its binary system of CuO@TiO_2_ heterojunction.

Photocatalyst	k_app_ (min^−1^)	t_½_ (min.)	E_EO_ (kWh/m^3^)	Q_app_. (%) (mol/Einstein)
TiO_2_	0.00909	77	769.2	0.782
CuO	0.01241	57.75	526.3	1.035
CuO@TiO_2_	0.03688	18.73	172.4	3.20

The low decomposition rates of CuO and TiO_2_ nanoparticles could be attributed to the fast recombination of e^−^/h^+^ pairs and inefficient quantum yield during the photocatalytic processes. The superiority of CuO@TiO_2_ binary system is due to the efficient charge transfer, enlarged light response range, and suppressed the photocarrier recombination that originated from the synergistic effects of two photocatalysts, TiO_2_ and CuO that resulted from p-n heterojunction between p-type CuO and n-type TiO_2_^53^. Photogenerated holes diffuse from CuO (p-type) to TiO_2_ (n-type) and electrons diffuse from TiO_2_ to CuO due to the existence of carrier concentration gradients. An electric field is formed at the junction when it is in the equilibrium^54^.

Photogenerated electrons can migrate the conduction band of TiO_2_ (n-type) and holes to the valence band of CuO (p-type) during photocatalysis. The p–n CuO@TiO_2_ heterojunction increases the photocatalytic activity by facilitating the separation of photogenerated electrons and holes. Additionally, the sensitization by CuO increased the optical absorption properties of TiO_2_ and improved charge carrier lifetime in the system that leads to enhancing the photocatalytic activity of CuO@TiO_2_. As indicated by XRD, HR-TEM, XPS, UV–Vis., PL, and EIS characterizations as well as DFT investigations, heterojunction formation considerably influenced the photocatalytic activity of CuO@TiO_2_ due to its influences on the photocatalyst microstructure and band structure.

### Quantum efficiency

The quantum yield can be detected by estimating the rate of disappearing of the reactant molecule or the formation of the product molecule divided by the photons absorbed per unit time, which can be used to quantify the heterogeneous catalysis. A considerable portion of the incident light is reflected or scattered by dispersed photocatalysts and is not absorbed by the dye solution. In most cases, there is no way to measure the amount of light absorbed by the photocatalyst experimentally. Another parameter frequently stated is the apparent quantum yield (Q_app_), which avoids the challenges of estimating the quantum yields in the photocatalytic reaction^55^.

Table 3 clarifies that Q_app_ for UV/CuO@TiO_2_ system is higher than that of the UV/TiO_2_ and UV/CuO systems. As a result of the poor quantum yield, CuO and TiO_2_ nanoparticles had the lowest photocatalytic activity.

### Investigations of E_EO_

The economic analysis of photocatalysis is critical factor that accounts for a large portion of operating costs. As a result, it is vital to evaluate the photocatalytic process' electrical energy consumption under experimental settings. Because it follows the pseudo first order kinetic model, the electrical energy per order (E_EO_) is a useful indicator of the photocatalysis process^56^. The E_EO_ enables a quick calculation of the cost of electrical energy and indicates the overall power needed. The treatment efficiency for the samples is evaluated using E_EO_ values for comparative study.

The E_EO_ values were calculated using the inverse of the slope of a plot of log (C_o_/C) versus UV dose (Fig. 11c). It was determined that the figure-of-merit method is appropriate for calculating the electrical energy efficiency. It is not only demonstrating the decline in the amount of electricity required by the photocatalytic system, but also the significant impact of the UV dose on the E_EO_ in the process. From Table 3, the E_EO_ values were established to be depending on the photocatalyst's nature. Furthermore, the E_EO_ values of the UV/CuO@TiO_2_ system are lower than those of the blank UV/TiO_2_ or UV/CuO system, indicating that the lower energy consumption is attributable to the higher applied potential and formation of highly reactive radical species. In summary, these insights can be used to design photocatalytic systems that use less electrical energy, have a higher rate constant, and cost less to operate.

### Role of reactive oxygen species

To get insights into the photodegradation mechanism of AR8 dye over the CuO@TiO_2_ binary system and understand the role of photo-generated holes and radicals in the photodegradation process, the charge trapping studies were conducted. To explore the role of reactive oxygen species (ROS), the photocatalytic activity of CuO@TiO_2_ was greatly suppresed by the addition of EDTA, K_2_Cr_2_O_7_, benzoquinone (BQ), and tert-Butyl alcohol (TBA) as model scavengers to capture holes (h^+^), electron (e^−^), superoxide radical (^**·**^O_2_^-^), and hydroxyl radical (^**·**^OH), respectively^57^. The AR8 degradation efficiency by CuO@TiO_2_ in the existence of various scavengers is shown in Fig. 11d. Both BQ and TBA had stronger suppressing effects on the photocatalytic degradation of AR8 than that for EDTA and K_2_Cr_2_O_7_, suggesting that ^**·**^O_2_^−^ and ^**·**^OH are the principal active species in the photocatalytic degradation of AR8 in the presence of a binary system of CuO@TiO_2_.

Based on the outcomes of the trapping tests, the following Eqs. (1–6) can be offered as a possible photodegradation mechanism of AR8 dye in the presence of CuO@TiO_2_ heterojunction.

1 $$ {\text{CuO}}@{\text{TiO}}_{{2}} + {\text{ h}}\nu \to {\text{e}}^{ - } + {\text{ h}}^{ + } , $$

2 $$ {\text{e}}^{ - } + {\text{ O}}_{{2}} \to {}^{ \cdot }{\text{O}}_{{2}}^{ - } , $$

3 $$ {\text{e}}^{ - } + {}^{{\mathbf{ \cdot }}}{\text{O}}_{{2}}^{ - } + {\text{ 2H}}^{ + } \to {\text{H}}_{{2}} {\text{O}}_{{2}} , $$

4 $$ {\text{2 e}}^{ - } + {\text{ HO}}_{{2}}^{{\mathbf{ \cdot }}} + {\text{ H}}^{ + } \to {}^{{\mathbf{ \cdot }}}{\text{OH }} + {\text{ OH}}^{ - } , $$

5 $$ {\text{h}}^{ + } + {\text{ OH}}^{ - } \to {}^{{\mathbf{ \cdot }}}{\text{OH,}} $$

6 $$ {\text{h}}^{ + } ,{\text{H}}_{{2}} {\text{O}}_{{2}} ,{}^{{\mathbf{ \cdot }}}{\text{O}}_{{2}}^{ - } ,{}^{{\mathbf{ \cdot }}}{\text{OH}} + {\text{ AR8}}\,{\text{dye}} \to {\text{ degradation}}\,{\text{products }}\left( {{\text{CO}}_{{2}} ,{\text{ H}}_{{2}} {\text{O}},{\text{ SO}}_{{4}}^{{ - {2}}} ,{\text{ NH}}_{{4}}^{ + } } \right). $$

### Stability and recyclability of CuO@TiO_2_ photocatalyst

**Figure 12:**
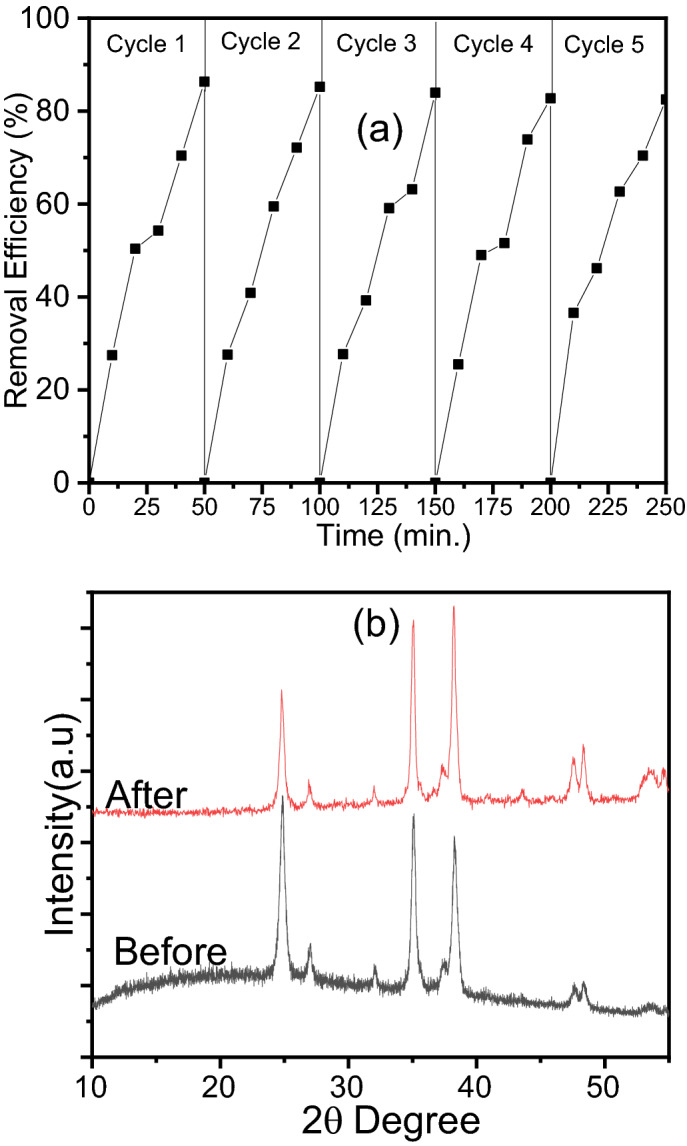
(**a**) Cycling runs, and (**b**) XRD before and after photocatalytic degradation of AR8 dye over CuO@TiO_2_.

From the economic point of view, the stability and good recyclability are two essential features for large-scale application of photocatalysis^58^. Using the same protocol, the degradation of AR8 were done on subsequent repeated cycles by reusing the CuO@TiO_2_ collected after each cycle. Then, the degradation percentage was calculated in each run. The stability and recyclability of CuO@TiO_2_ towards AR8 dye photodegradation were evaluated after five consecutive runs and the obtained data are presented in Fig. 12a. It is clear that the reduction in the removal efficiency was negligible (< 4.5%), which denotes the nature of high stability after 5 cycles. Wherefore, CuO@TiO_2_ is an economically suitable photocatalyst for industrial application from a practical point of view.

To evaluate the stability of CuO@TiO_2_ photocatalyst, the XRD patterns were compared before and after the first cycle of the photocatalytic degradation of AR8 dye as shown in Fig. 12b. Obviously, the maintained structure after photocatalysis was evidenced by the unchanged XRD reflections. All peaks corresponding to the interpretation based on Fig. 1a, represent the stability of CuO@TiO_2_.

As it is known, the porous structure is one of the most important requirements for the ideal photocatalysis process. Table 4 shows the textural nature of sample CuO@TiO_2_ and its recycled sample after photocatalysis that was investigated by BET analysis (Fig. 5). There is a slight decrease in the surface area after recycling indicating low structural changes occurrence by recycling (Table 4). The pore volume and pore diameter were also slightly reduced. Even after the recycling, the catalyst still had a feasible pore diameter that can accommodate the flow of reactant intermediate molecules) through the photocatalyst during the reaction.*S*_*BET*_ surface area based on BET equation, *S*_*mico*_ micropores surface area, *L*_*0*_ micropore width, *V*_*T*_ total pore volume, *V*_*meso*_ mesopores volume.

**Table 4:** Textural characteristics of the CuO@TiO_2_ before and after photocatalysis.

Samples	S_BET_ (m^2^ g^−1^)	S_micro_ (m^2^ g^−1^)	L_o_ (nm)	V_T_ (cm^3^ g^−1^)	Pore diameter (nm)	V_meso_ (cm^3^ g^−1^)
Before	19	34	3.52	0.0312	6.08	0.007
After	18	32	3.48	0.0291	6.01	0.006

**Figure 13:**
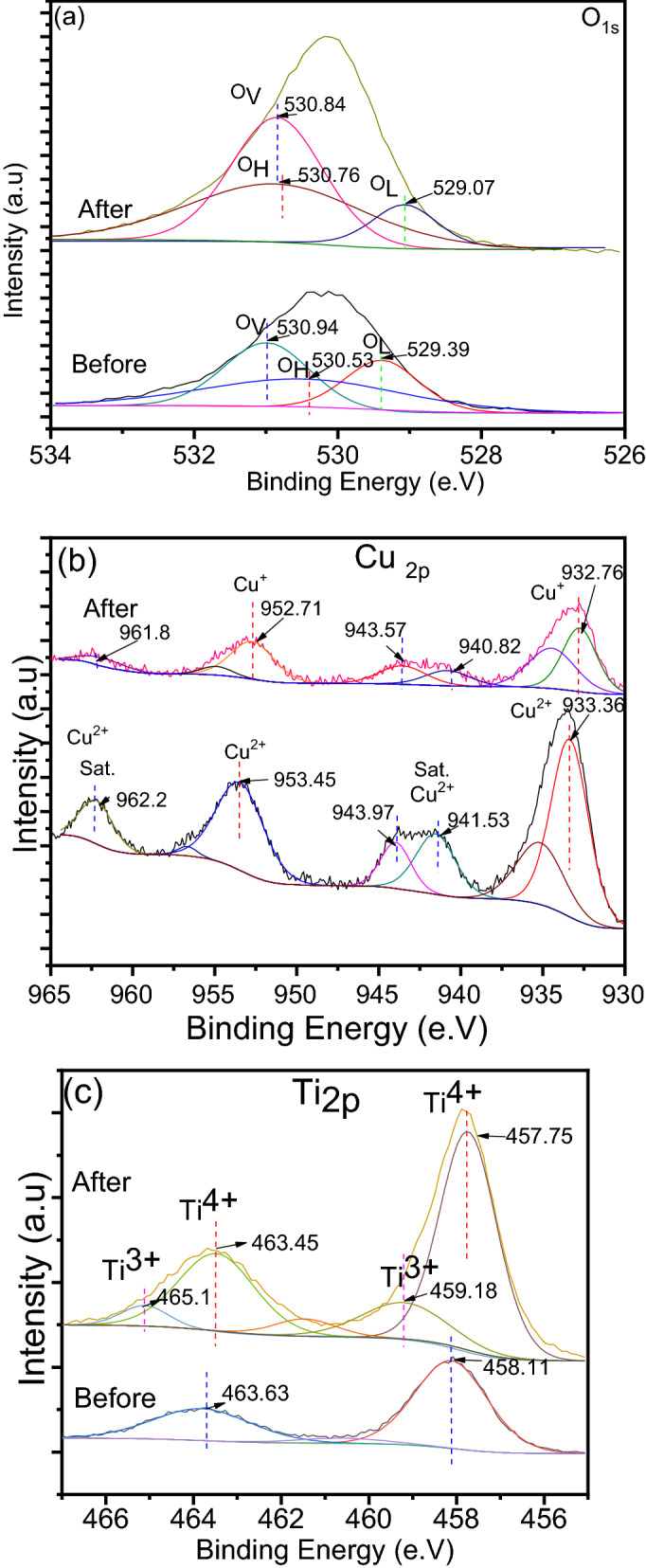
XPS of (**a**) O1s, (**b**) Cu2p, and (**c**) Ti 2p of CuO@TiO_2_ before and after photocatalysis.

To discuss the mechanism of the photocatalytic reaction for CuO@TiO_2_, an XPS analysis was investigated the elemental valence states on the surface of CuO@TiO_2_ before and after AR8 removal. The changes of element state before and after the photocatalytic reaction are revealed in Fig. 13 and Table 5. Figure 13a shows that the O_1s_ peaks of CuO@TiO_2_ were divided into three peaks. After photocatalysis, the peak at 529.39 eV belonged to the lattice oxygen (O_L_), including Ti–O and Cu–O, was shifted to 529.07 e.V^29^. Also, the peak at 530.94 e.V attributed to ions O_2_^−^ staying in the oxygen vacancy (OV) that is shifted to 530.84 e.V. The peak at 530.53 eV was attributed to hydroxyl oxygen (O_H_) that shifted to 530.76 e.V after photocatalysis. The relative ratios of elements for catalysts have been calculated and are shown in Table 5. The oxygen content in TiO_2_ and CuO mainly composed of O_L_, and the proportion of O_H_ and O_V_ is very small (Table 1), which reduced to 24.36% in the composite. After photocatalysis, the concentration of O_L_ is reduced to 10.02%, which confirmed the contribution of O_H_ and O_V_ in the photocatalytic process (Table 5) and supported the results of the role of reactive oxygen species (Fig. 11d) and photodegradation mechanism (Eqs. 1–6). Furthermore, CuO@TiO_2_ displayed lower O_H_ than before photocatalysis, which was ascribed to the high oxidation ability of the CuO@TiO_2_ structure (Table 5). Also, CuO@TiO_2_ displayed much higher oxygen vacancy (Ov/O) than before photocatalysis, which was attributed to the greater loss of lattice oxygen by TiO_2_ nanoparticles to introduce more oxygen vacancies to the composite (Table 1).

**Table 5:** The relative oxygen content for CuO@TiO_2_ before and after photocatalysis.

Sample	O_L_/O	O_H_/O	O_V_/O
Before	24.36	39.77	35.87
After	10.02	35.26	54.71

After UV illumination, Fig. 13b show the peaks of Cu 2p_3/2_ and Cu 2p_1/2_ moved to 932.76 and 952.71 eV, respectively, being assigned to Cu^+^^59^. These revealed that most of surface Cu^2+^ was reduced to Cu^+^ upon the UV irradiation^60^. The photocatalytic process introduced more oxygen vacancies on the surface of CuO@TiO_2_, which is reflected in Table 5. Under UV light irradiation, the electron can transfer from the valence band to the surface CuO through interface charge transfer, leading to the reduction of Cu^2+^ to Cu^+^^3^.

After photocatalysis, the 457.75 and 463.75 e.V positions were assigned to typical Ti 2p _3/2_ and Ti 2p _1/2_ peaks in CuO@TiO_2,_ respectively, which indicate there is a small shift of all peaks when compared to the composite before photocatalysis (Fig. 13c). This shift of binding energy values is attributed to the redistribution of electric charge in the composite materials^61^. In addition, the spectra of Ti 2p region showing the presence of Ti^3+^ that formed from the reduction of Ti^4+^ and denoting the influence of UV light on the nature of the Ti phase, in agreement with TiO_2_ results previously reported^28^. Despite the certain reduction observed the difference in BE between Ti 2p3/2 and Ti 2p1/2 components are always maintained at 5.7 eV.

### Structure activity correlation and proposed photocatalytic mechanism

**Figure 14:**
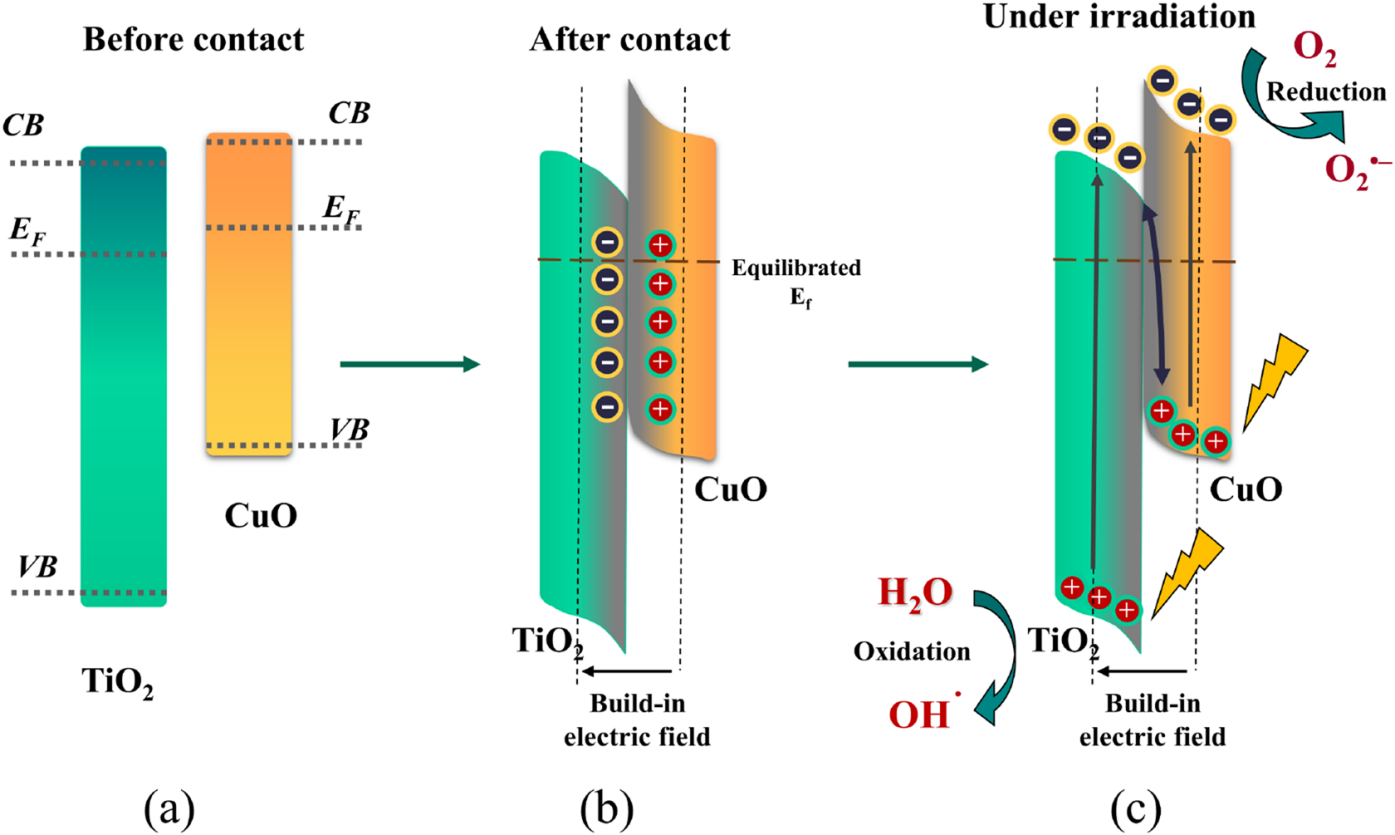
Schematic illustration of the S-scheme transfer mechanism between TiO_2_ and CuO (**a**) Before contact, (**b**) After contact, and (**c**) After contact in light. *CB* conduction band, *VB* valence band, *E*_*F*_ fermi level.

The plausible mechanism of the photocatalytic degradation of AR8 dye over CuO@TiO_2_ heterojunction composite is proposed in Fig. 14 based on the aforementioned experimental findings and characterization analysis. In this system, upon direct irradiation, the e^−^/h^+^ pairs would be formed on both TiO_2_ and CuO. In the transfer process, the lifetime of the excited electrons and holes is extended, resulting in better quantum efficiency (Table 3).

Furthermore, this considerably improves the separation of the photogenerated electron–hole pairs and limits their quick recombination, resulting in CuO@TiO_2_ having a higher photocatalytic activity than TiO_2_^56^. When the combination of TiO_2_ with CuO, an inner electrical field forms at the interface lead to improving the electron–hole separation^62^.

Because of TiO_2_ of lower EF and connected with CuO of higher EF, the CuO@TiO_2_ heterojunction is formed. However, the electrons will transfer from CuO to the TiO_2_ easily until the EF at the interface tends to equilibrate. Concurrently, CuO shows downward interface band bending and is positively charged at the interface owing to loss of electrons; while TiO_2_ shows upward interface band bending and is negatively charged at the interface owing to an accumulation of electrons. Meanwhile, the electrons transfer creates an internal electric field at interfaces pointing from CuO to TiO_2_. When the CuO@TiO_2_ heterojunction is exposed to light, under the combined effect of an internal electric field, interface band bending and Coulomb interaction, the photogenerated electrons in CB of TiO_2_ easily transfer to VB of CuO and recombine with the photogenerated holes of CuO. Meanwhile, the photogenerated holes on VB of TiO_2_ and the photogenerated electrons on CB of CuO are maintained, which participate in the photocatalytic redox reaction, respectively^63–66^.

Because CuO's conduction band is more negative than TiO_2_'s, photo-generated electrons have an affinity to transfer from CuO's conduction band to TiO_2_'s conduction band because of the difference in potential, reducing the possibility of photoinduced e^−^/h^+^ recombination and thus facilitating reduction reactions for the destruction of pollutants^67^. Meanwhile, because TiO_2_'s VB is more positive than CuO's, the holes transfer from TiO_2_'s VB into CuO's VB, which carry out oxidation reactions with water to form a proton and intermediate products. This causes the photogenerated electrons to be accumulated at the conduction band of TiO_2_ and also to be accumulated of holes at the valence band of CuO. As a result of the higher conduction and valence band of CuO than TiO_2_, the transfer process is thermodynamically advantageous^68,69^. Hence, the e^−^/h^+^ pair recombination rate in the CuO@TiO_2_ is substantially lower than in pure TiO_2_. Furthermore, the electrons help Ti^3+^ ions in having a longer lifetime and improve ions transference at the interface between CuO and TiO_2_. Overall, the p–n heterojunction may prevent the recombination of the photogenerated electrons from recombining with holes.

According to the abovementioned results of the effect of reactive oxygen species and the results from XPS before and after photocatalysis, the ongoing electrons reduced dissolved oxygen, generating ^**·**^O_2_^−^ (Eq. 2) and the following ^**·**^OH (Eq. 4) which was the governing active species for the pollutant attacking that consistent with the results of effect of scavengers (Fig. 11d). On the other hand, the holes stay in the VB of CuO that helps in the conversion of OH^−^ to ^**·**^OH which leads to the effective degradation of AR8 dye by the direct oxidation of photogenerated holes. The AR8 dye can be destroyed in two ways. (i) in the VB of CuO, the conversion of hydroxyl anion (OH^−^) to hydroxyl radical (^**·**^OH) (Eq. 5); and (ii) the formed electrons from the CB of TiO_2_ are transferred to the adsorbed oxygen (O_2 adsorbed_) for generation of superoxide anion radical (^**·**^O_2_^−^) (Eq. 2). Continuous production of highly strong oxidants (^**·**^O_2_^−^ and ^**·**^OH) leads to the oxidation AR8 to CO_2_ and H_2_O^21^.

According to the thoughts presented above, the highly boosted photocatalytic activity of CuO@TiO_2_ heterostructures compared to pure TiO_2_ can be attributed to a number factors, including the intensification in the photo-absorption, perfection in the charge separation efficiency and direct oxidation of AR8 dye with CuO after the construction of p–n heterojunctions. The synergistic impact (for example, heterojunction-induced effects on CuO@TiO_2_ photocatalysts) can aid to strengthen the separation of photo-generated electrons and holes (Fig. 14). As a result, the CuO@TiO_2_ composites outperform ordinary TiO_2_ in terms of photocatalytic activity. Another benefit for photocatalytic decomposition by CuO@TiO_2_ heterojunction nanocomposite is to form electrons that help Ti^3+^ ions for extending the lifetime and improve the transfer of electrons at the p–n junction which suppresses the electron/hole recombination.

## Materials and chemicals

TiO_2_ (Degussa P 25, average particle size about 30 nm, 70% anatase form and of surface area about 50 m^2^ g^−1^) was purchased from Degussa company, Germany. CuCl_2_·2H_2_O, NaOH and glucose were purchased from Aldrich. All aqueous solutions were prepared using distilled water from the Millipore instrument. Acid red 8 dye with molecular formula C_18_H_14_N_2_Na_2_O_7_S_2_, and molecular weight of 480.42 g/mol, with λ_max_ 508 nm was purchased from Fluka.

### Synthesis of photocatalysts

CuO nanoparticles were prepared by free-template method by dissolving 1 g of copper precursor in 50 mL solvent (water/ethanol) under a constant stirring at room temperature. A precipitate was produced when NaOH solution (3 M, 10 mL) is added dropwise to the above solution. After being stirred for 5 min, d-glucose powder (0.2 g) was added into the dark precursor and the temperature was raised up to 70 °C with stirring for 15 min. The precipitate gradually turns into brick red and then it was allowed to cool to room temperature and the obtained precipitates were centrifuged. The precipitate was allowed to centrifuge twice more in de-ionized water and anhydrous ethanol, respectively. Finally, the precipitates product was dried at 50 °C overnight.

The S-scheme CuO@TiO_2_ heterojunction nanocomposite was prepared through a solid-state reaction route. 50 wt. of Cu_2_O nanoparticles were added to 50 wt.% TiO_2_ which have been mixed uniformly and made fine by grinding in ball mill for 2 h to get fine nanopowders. The resultant powders were calcined in air at 500 C for 3 h in an electric muffle furnace.

### Correlation between physicochemical characteristics and photocatalytic activity

The detailed of physicochemical characterizations (S-1), evaluation of photocatalytic activity (S-2), intensity measurements (S-3), degradation kinetic (S-4), recycling and stability test (S-5), evaluation of apparent quantum yield (Q_app_) and electrical energy per order (E_EO_) (S-6), detection of reactive species (S-7), electrochemical impedance spectroscopy (EIS) (S-8), and computational methodology (S-9) are stated in supporting information.

## Conclusions

To the best of our knowledge, this is the first time we have shown that the fabricating a binary system of S-scheme CuO@TiO_2_ heterojunction composite photocatalyst via a simple solid-state reaction is a promising strategy for boosting the photocatalytic degradation of toxic organics. The existence of heterojunction between TiO_2_ and CuO was demonstrated by the measurements of catalytic activity, including HRTEM, Raman, XPS, UV–Vis. PL and EIS. Also, the experimental optical results showed that the CuO@TiO_2_ reduced the band gap by shifting the band-gap of commercial TiO_2_ (3.43 eV) into a new band gap of CuO@TiO_2_ (2.35 e.V) and DFT calculation explained that by details. The S-scheme CuO@TiO_2_ heterojunction composite, displaying the lowest PL intensity, shows the highest activity, and reveals the apparent quantum efficiency of 3.2%. The higher photocatalytic activity was qualified to the well-designed S-scheme CuO@TiO_2_ heterojunction, which remarkably aids the separation of photogenerated electrons and holes by the synergistic impacts at the interface of the catalyst components. Further, the most active species in the photodegradation of AR8 dye was detected as ^**·**^OH and ^**·**^O_2_^−^. However, the experimental results demonstrated that the h^+^, and e^−^ are also involved in the degradation process. The plausible degradation mechanism by p–n S-scheme CuO@TiO_2_ heterojunction was determined and discussed based upon detection of reactive oxygen species, PL and EIS, which found that the the electron transfer from CuO to TiO_2_, followed by hole transfer from TiO_2_ to CuO was attributed to be the most likely mode of charge transfer in the S-scheme CuO@TiO_2_ system. The recycling experiment revealed that the AR8 dye is diminished by 4.5% after five runs of CuO@TiO_2_. The result certified the suitable stability of CuO@TiO_2_ for long-term applications. Further theoretical work is currently underway to investigate the formation of S-scheme CuO@TiO_2_ heterojunction. Potentially, this study offers a new way for the strategy of the highly efficient S-scheme heterojunction photocatalyst for the effective degradation of organic pollutants in large scales.

## Supplementary Information

Supplementary Information.

## Data Availability

All data generated or analyzed during this study are included in this article (and its Supplementary Information file).
