# Supplementary material for: The superior photocatalytic performance and DFT insights of S-scheme CuO@TiO2 heterojunction composites for simultaneous degradation of organics
Source: Sci Rep. 2022 Feb 9;12:2217. doi: 10.1038/s41598-022-05981-7 (PMC8828870; doi:10.1038/s41598-022-05981-7)
Supplement: Supplementary file 1 — Supplementary Information. [file 41598_2022_5981_MOESM1_ESM.docx]

**Supplementary information**

**The superior photocatalytic performance and DFT insights of S-scheme CuO@TiO_2_ heterojunction composites for simultaneous degradation of organics**

**Hesham Hamad ^a,*^,** **[Mohamed M. Elsenety](https://www.sciencedirect.com/science/article/abs/pii/S1387700320308030#!) ^b^, Wagih Sadik ^c^, Abdel-Ghaffar El-Demerdash ^c^ Adel Nashed ^c^, Amr Mostafa ^c^, Shaimaa Elyamny ^d^**

^a^ Fabrication Technology Research Department, Advanced Technology and New Materials Research Institute (ATNMRI), City of Scientific Research and Technological Applications (SRTA-City), New Borg El-Arab City, 21934, Alexandria, Egypt.

^b^ Department of Chemistry, Faculty of Science, Al-Azhar University, P.O. 11823, Nasr City, Cairo, Egypt

^c^ Materials Science Department, Institute of Graduate Studies and Research (IGSR), Alexandria University, Alexandria, Egypt.

^d^ Electronic Materials Research Department, Advanced Technology and New Materials Research Institute, City of Scientific Research and Technological Applications (SRTA-City), New Borg El-Arab City, P.O. Box 21934, Alexandria, Egypt

* Corresponding author: Hesham A. Hamad ([hhamad@srtacity.sci.eg](mailto:hhamad@srtacity.sci.eg) , [heshamaterials@hotmail.com](mailto:heshamaterials@hotmail.com))

***S-1 Physicochemical characterizations***

The crystal phases of catalysts were measured using X-ray diffractometer (Shimadzu 7000, Japan) at a wavelength of 1.54 Å using K_α_ of Cu radiation. The surface morphology and elemental characterization of sample of CuO@TiO_2_ was studied using a scanning electron microscope (SEM, JEOL JSM 6360 LA, Japan) and a high-resolution transmission electron microscope (HR-TEM, JEOL 2100, Japan) that integrated with energy-dispersive X-ray spectroscopy. Raman spectra were recorded at different locations of the sample using a SENTERRA (spectrometer- Bruker, Germany) with a 532 nm Ar laser. The textural properties of the CuO@TiO_2_ before and after photocatalysis process were obtained by N_2_ sorption isotherms method at −196°C using Microtrac-BEL, Japan. The specific area was determined by Brunauer-Emmett-Teller (BET) method, while the pore distribution was obtained using Brunauer- Joyner-Halenda (BJH) equation. X-ray photoelectron spectroscopy (XPS) spectra of C_1s_, O_1s_, Cu_2p_, and Ti_2p_ regions of CuO@TiO_2_ before and after photocatalysis were obtained on a Kratos Axis Ultra-DLD X-ray photoelectron spectrometer (Kratos Analytical Ltd., Kyoto, Japan). The UV-Vis. spectra were recorded using a spectrometer (Shimadzu model 1601 PC double beam spectrophotometer, Japan). The photoluminescence spectrum was recorded on (Agilent Technologies, United States) with 50 W Xe lamp steady state excitation source with a 360 nm excitation wavelength and 400 nm filter.

***S-2. Evaluation of photocatalytic activity***

The photocatalytic performance of the prepared photocatalysts was used to evaluate the removal efficiency of AR8 dye in an aqueous solution using batch slurry photoreactor. The contents (TiO_2_/dye, CuO/dye, and CuO@TiO_2_/ dye) of the glass container were agitated by a magnetic stirrer and kept purged with air (rate 3000 ml min.^-1^). The dye solution was agitated with photocatalyst for adsorption at 30 min before irradiation with UV to obtain equilibrium adsorption.

Irradiation was done with a tubular low-pressure mercury lamp with power 43 W and wavelength 254 nm (Voltarc Tubes Inc., USA). The color disappearing of AR8 dye was analyzed spectrophotometrically at its maximum absorption wavelength of 508 nm, using Shimadzu model 1601 PC double beam spectrophotometer, Japan. Samples containing CuO@TiO_2_ were taken periodically from the photoreactor and measured after filtration using 0.2 µm polyethersulfone membrane. The efficiency of the photocatalytic activity can be estimated in equation 1.

% Degradation rate = [Co – C] / Co × 100% = [Ao – A] / Ao × 100% (1)

Where Co = initial concentration of dye solution (mg/L), C = concentration of dye solution after photoirradiation (mg/L) at time t (minute). Ao is the value of absorbance of dye aqueous solution after adsorption in the dark, and A is the value of absorbance of dye aqueous solution after reaction.

***S-3. Intensity measurements***

Irradiation was carried out with a tubular low pressure mercury lamp (total rating 43 W, total UV output at 254 nm 13.4 W, and length 120 cm, Voltarc Tubes Inc., USA) that is located 10 cm apart from the surface of the dye solution. The total intensity reaching the slurry solution was measured using a UVX radiometer (UV Products Ltd., Cambridge, UK) equipped with a sensor with peak sensitivity at 254 nm was 4 mWcm^-2^.

***S-4. Degradation kinetic***

The decomposition kinetics of AR8 dye by TiO_2_, CuO, and its composite of CuO@TiO_2_ were assessed by following the Langmuir-Hinshelwood model. When the chemical concentration Co is millimolar solution the integrated form of equation (2) be an apparent first order equation.

ln (C_o_/C) = k_app_ t (2)

Where, k_app_ represents the apparent first order rate constant, C_o_ and C are concentration before and photocatalytic reaction, respectively. The half-life time (t_½_) of the first order reaction is the time required for the reactants to be degraded to the half of their C_o_. The relationship between t_½_ and k_app_ is given by equation (3).

t_½_ = 0.693 / k_app_ (3)

***S-5. Recycling and stability test***

The recycling of CuO@TiO_2_ was carried out as follows: following the first photodegradation cycle, the treated AR8 dye solution was filtered to remove the photocatalyst. The photocatalyst was then separated for reuse and dried in a 60 ^o^C oven for 12 hours. The separated photocatalyst was reintroduced into the next cycle, and the process was done five times to ensure the photocatalyst's stability. XRD, XPS, and BET were used to characterize the recycled photocatalyst.

***S-6. Evaluation of apparent quantum yield (Q_app_) and electrical energy per order (E_EO_)***

The estimation of quantum yield as follows;

Apparent quantum yield (Q_app_*,* mol */* Einstein) = k_app_ C_o_ / I (4)

Where k_app_ is the apparent first-order rate constant, C_o_ is the initial dye concentration and I is the total intensity of incident photons entering the reactor cell.

Considering first-order degradation kinetics, the UV doses were calculated for all photocatalysts using eq. 5. From the UV doses, the simplest form of the estimation of E_EO_ can also be calculated using eq. 6.

UV Dose = [Lamp power (kW) × Time (h) × 1000] / [Treated volume (L)] (5)

E_EO_ **=** UV Dose / [Log (C_o_/C)] (6)

***S-7.Detection of reactive species***

The reactive species detection process is similar to the photodegradation experimental process. Various scavengers were subjected into the AR8 dye solution prior to addition of photocatalyst. The tertiary butyl alcohol (TBA), benzoquinone (BQ), EDTA, and K_2_Cr_2_O_7_ were used as ^●^OH, ^●^O_2_^-^, h^+^, and e^-^ scavengers, respectively.

***S-8. Electrochemical impedance spectroscopy (EIS)***

The electrochemical impedance spectroscopy (EIS) was performed at room temperature using a computer-controlled Potentiostat (Metrohm Autolab, model: 87070). 9 mg from the final product powders and 1 ml ethanol were mixed by sonication for 20 min to form uniform suspension. Then, 100 μL suspension was dropped onto an ITO glass (1.0 cm × 1.0 cm) by a dropper. The samples were dried at 100 °C for 12 h to obtain working electrodes. We employed a standard three electrode configuration, in which the ITO, Pt foil, and a saturated calomel electrode were used as the working, counter and reference electrodes, respectively. The electrochemical reaction was carried out in an aqueous electrolyte containing 0.5 M Na_2_SO_4_. The tests were recorded in a frequency range from 0.01 to 10^6^ Hz with a sinusoidal signal of 5 mV.

***S-9. Computational methodology***

The theoretical calculations were carried out using CASTEP code in the in gas phase, in order to estimate the electronic band structure, partial density of states (PDOS), and charge energy distributions, Based on the plane-wave pseudopotential method of quantum density functional theory (DFT).^1^ The GGA/PBESOL exchange-correlation model was used to study the electron-ion interaction with cut-off energy equal to 480 eV for all samples. However, to obtain well-relaxed structures, geometry optimization was carried out using the BFGS algorithm. ^2,3^

**Figure S1: High-resolution XPS scan of O1s of (a) TiO_2_, (b) CuO, and (c) CuO@TiO_2._**

**References:**

1. Segall, M.D., Lindan, P.J.D., Probert, M.J., Pickard, C.J., Hasnip, P.J., Clark, S.J., Payne, M.C. First-principles simulation: ideas, illustrations and the CASTEP code. *J. Phys. Condens. Matter.* **14** ,2717–2744 (2002).

2. Hernández-Haro, N., Ortega-Castro, J., Martynov, Y.B., Nazmitdinov, R.G., Frontera, A. DFT prediction of band gap in organic-inorganic metal halide perovskites: An exchange-correlation functional benchmark study. *Chem. Phys.* **516**, 225–231 (2019).

3. Clark, S.J., Segall, M.D., Pickard, C.J., Hasnip, P.J., Probert, M.I.J., Refson, K., Payne, M.C. First principles methods using CASTEP, Zeitschrift Fur Krist. **220**, 567–570 (2005).
